# Maritime Provinces Staphylinidae (Coleoptera): Addenda and Corrigenda

**DOI:** 10.3897/zookeys.126.1778

**Published:** 2011-09-02

**Authors:** Christopher G. Majka, Jan Klimaszewski

**Affiliations:** 1 Nova Scotia Museum, 1747 Summer Street, Halifax, Nova Scotia, Canada B3H 3A6; 2 Natural Resources Canada, Canadian Forest Service, Laurentien Forestry Centre, 1055 rue du P.E.P.S., PO Box 10380, Stn. Sainte-Foy, Québec, QC, Canada G1V 4C7

##  

Majka and Klimaszewski (2010) surveyed the Aleocharinae fauna of the Maritime Provinces of Canada, reporting that 203 species were known in the region. They also added 16 new provincial records from the region. Inadvertently, they neglected to provide new provincial records of three species that were indicated as occurring in Nova Scotia in Table 1 (pp. 23-33) of their results. This omission is rectified below wherein *Gyrophaena modesta* Casey, *Gyrophaena subnitens* Casey, and *Placusa vaga* Casey are all newly recorded as occurring in Nova Scotia. Klimaszewski et al. (2010, pp. 77) also erroneously reported *Tachyporus nitidulus* as occurring in Prince Edward Island. There are no records of this adventive Palaearctic species from the province, although it is more widely distributed in Nova Scotia than hitherto reported. Additional records of *Tachyporus nitidulus* are provided from Nova Scotia.

***Gyrophaena modesta* Casey, 1906**

**NOVA SCOTIA: Kings County:** Wolfville, 19 September 1998, J. Ogden, sweeping grasses (1, J. Ogden coll.).

**Figure 1.**
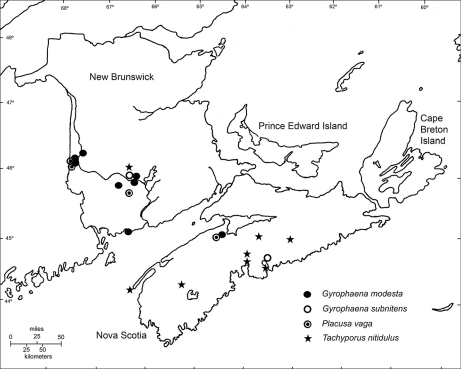
The distribution of *Gyrophaena modesta*, *Gyrophaena subnitens*, *Placusa vaga* and *Tachyporus nitidulus* in the Maritime Provinces of Canada. Localities indicated are from Klimaszewski et al. (2009), Webster et al. (2009), Majka and Klimaszewski (2008a), and the present study.

*Gyrophaena modesta* is newly recorded from Nova Scotia (Fig. 1). It was reported from Alberta and New Brunswick by Gouix and Klimaszewski (2007). It has also been found in the United States in Illinois, Indiana, Michigan, Minnesota, New Hampshire, and New York (Seevers 1951). It has been found in various coniferous, deciduous and mixed forests on fresh gilled fungi, and occasionally on decaying gilled fungi and *Pleurotus* sp. fungi on a log (Klimaszewski et al. 2009).

***Gyrophaena subnitens* Casey, 1906**

**NOVA SCOTIA: Halifax County:** Waverly, 14 May 1965, 27 May 1965, 8 June 1965, B. Wright, red oak, window trap (7, Nova Scotia Museum).

*Gyrophaena subnitens*is newly recorded from Nova Scotia (Fig. 1). It was reported from Manitoba and Ontario by Gouix and Klimaszewski (2007) and Seevers (1951), and from New Brunswick by Klimaszewski et al. (2009). It has also been found in the United States in Illinois, Kansas, Maine, Michigan, Minnesota, Missouri, New York, and Wisconsin (Seevers 1951). It has been recorded on gilled mushrooms in a mixed forest and in red oak (*Quercus rubra*) forest (Klimaszewski et al. 2009). In Nova Scotia it was collected in a red oak forest.

***Placusa vaga* Casey, 1911**

**NOVA SCOTIA: Kings County:** North Alton, 2 June 2005, D.H. Webster, under bark of *Populus tremuloides* windfall (1, DH Webster coll.).

*Placusa vaga* is newly recorded from Nova Scotia (Fig. 1). The specimen collected in North Alton was found in association with *Carpophilus sayi* Parsons (Nitidulidae) (abundant) and *Corticeus tenuis* (LeConte) (Tenebrionidae) (infrequent). *Placusa vaga* was reported from British Columbia and Québec by Gouix and Klimaszewski (2007) and from New Brunswick by Webster et al. (2009). Specimens in New Brunswick were also collected under the bark of poplars and at a sap flow on a recently cut poplar, as well as in drift material on a river margin.

***Tachyporus nitidulus* (Fabricius, 1781)**

**NOVA SCOTIA: Halifax County:** Halifax, 19 June 2009, S. MacIvor, open area, pitfall trap (1, Saint Mary’s University); **Hants County:** Upper Rawdon, 24 June 2008, 25 June 2008, 26 June 2009, 27 June 2009, 28 June 2009, 21 July 2009, 22 July 2009, 24 July 2009, 13 August 2009, 14 August 2009, 26 August 2009, 8 September 2009, J. Renkema, highbush blueberry field, pitfall trap (21, Dalhousie University); **Queens County:** Kejimkujik National Park, 24 August 1994, B. Wright, hemlock forest, leaf litter (1, Nova Scotia Museum).

*Tachyporus nitidulus* was reported from New Brunswick by Klimaszewski et al. (2005), and from Nova Scotia by Majka and Klimaszewski (2008a). Majka and Klimaszewski (2008b) discussed its zoogeographic status, noting (after Campbell 1979) that it may consist of two distinct species or populations. Pending further investigation, they regarded *Tachyporus nitidulus* as an adventive Palaearctic species in North America.

Subsequently, however, Klimaszewski et al. (2010) indicated that this species had also been found in Prince Edward Island. This reference, however, was incorrect. There are no collections or published reports of this species from the province. Consequently, *Tachyporus nitidulus* is removed from the faunal list of Prince Edward Island. It has, however, been found more widely in Nova Scotia than previously reported, and the records above provide additional collecting localities in the province (Fig. 1).

It is often found in moist habitats such as river debris and swampy areas, along streams, lakes, or in wet seepages. It is common in all kinds of rotting materials; found in leaf litter, decaying material in hollow logs and stumps, and in mammal nests; and has been swept from flowers and bushes (Campbell 1979).
